# A modular system of flexible receive-only coil arrays for 3 T Magnetic Resonance Imaging

**DOI:** 10.1016/j.zemedi.2023.05.002

**Published:** 2023-05-29

**Authors:** Lena Nohava, Michael Obermann, Roberta Frass-Kriegl, Onisim Soanca, Elmar Laistler

**Affiliations:** High Field MR Center, Center for Medical Physics and Biomedical Engineering, Medical University of Vienna, Vienna, Austria

**Keywords:** Flexibility, Magnetic Resonance Imaging (MRI), Modularity, Radio frequency (RF), Coil, Versatility

## Abstract

Flexible form-fitting radiofrequency coils provide high signal-to-noise ratio (SNR) for magnetic resonance imaging (MRI), and in array configuration large anatomical areas of interest can be covered. We propose a modular system - “ModFlex”- of flexible lightweight 4-channel coaxial coil arrays for 3 T MRI. We investigated the performance difference between commercial reference coils and 8- and 16-channel ModFlex receive-only array systems. In vivo, six anatomical targets in four regions of interest – the neck, the ankle, the spine and the hip – were imaged with the novel coil array system. The versatility of ModFlex and the robustness of the coil characteristics for different use cases is demonstrated. We measured an SNR gain for 4 out of 6 and similar SNR for 2 out of 6 anatomical target regions as compared to commercial reference coils. Parallel imaging capabilities are comparable to standard coils in hip and neck imaging, but ModFlex outperforms standard coils in ankle and spine imaging. High SNR combined with high acceleration possibilities enables faster imaging workflows and/or high-resolution MR acquisitions. The coil’s versatility is beneficial for use cases with varying subject sizes and could improve patient comfort.

## 1 Introduction

Radiofrequency (RF) coils are the components in Magnetic Resonance Imaging (MRI) responsible for MR signal generation and reception. In recent years, 3 Tesla has become the clinical standard for ^1^H MRI, slowly replacing 1.5 T in developed countries [1]. At 3 T, depending on the imaging application, optimized receive-only coils are typically employed in combination with the scanner-integrated transmit body coil. High achievable signal-to noise ratio (SNR) of receive coils allows for imaging with high spatial resolution, and together with a suitable spatial distribution of coil elements depending on the application and low noise correlation between coils, high SNR is a prerequisite for a scan time reduction using parallel imaging techniques [2], [3]. SNR is increased by placing the receive coil as close as possible to the sample, i.e., the target area of the human body. This maximizes the electromagnetic coupling between coil and sample, and thus, the induced voltage in the receive coil which translates to higher achievable SNR. Considering signal detection and relevant noise sources (i.e., mostly sample and internal coil noise), as a rule of thumb, for circular surface loop coils, an SNR optimum is given if the coil diameter is approximately equal to the desired penetration depth [4]. The use of coils in array configuration [5] allows to combine the high SNR of local coil elements with a large field-of-view and enables parallel imaging. Taken together, these aspects motivate the design of radiofrequency coil arrays which have a form-fitting design with an overall array geometry and individual element size adapted to the target anatomy. The majority of MR imaging scenarios are limited to a certain body part, i.e., a specific field-of-view (FOV), in contrast to other imaging approaches (e.g. computer tomography or X-ray) due to the duration and related cost of a high-quality whole-body MR exam. Currently, multiple dedicated coils for each application are used with a large part of them being mechanically rigid. Some commercial coils feature the option of deactivating certain elements not useful for imaging (e.g., spine or head/neck coil provided by Siemens Healthineers, Erlangen, Germany) but without the knowledge of the exact coil geometry, this task can be difficult. Coil sensitivity outside of the desired FOV can lead to unwanted fold-over artifacts. Therefore, limiting the coil sensitivity to the target FOV motivates the concept of modular form-fitting coil arrays.

Recent advances in RF coil technology [6] have been centered around form-fitting coil design. Existing technologies include flexible coils proposed by MR system vendors (“AIR™ coils”, GE Healthcare, Waukesha, WI, USA or “Contour coils”, Siemens Healthineers, Erlangen, Germany) or coils developed in research projects, especially highlighting the usability of flexible transmission line resonator designs [7], [8], [9], [10], [11], [12]. Our group has proposed coaxial transmission line resonators (“coaxial coils”) comparable to the first implementation of shielded loop resonators in MRI by Zabel *et al*. [13], developments done by Zhang *et al*. [14] (“high impedance coils”) or designs described in the patent by Yang *et al*. [15]. Coaxial coils are an interesting technology for the fabrication of flexible receive coil arrays at 3 T for several reasons: Proposed designs [8], [14] come without any lumped elements along the coil conductor, suitable coaxial cables are very thin (≈1 mm) and ultra-flexible [8]. Furthermore, the coil performance is robust against small bending or elongation in different use cases [10], [14], [16], [17]. Strategies to optimize the interfacing circuitry of coaxial coils regarding SNR and inter-element decoupling performance [18] have been demonstrated, and it has been shown that the printed circuit board and housing can be miniaturized [16]. The miniaturization aspect is especially relevant for coaxial coils to maintain mechanical flexibility when interfacing the coil to the scanner whereas for rigid coils in a bulky housing this aspect is often not that critical, e.g. with more free housing space for circuit boards, larger components or cables.

In other work, stretchable coil designs [19], [20], [21], [22], [23] have been investigated which can be form-fitted to the subject’s body. However, with stretchable coils, the added difficulty are significant resonance frequency shifts due to coil inductance or capacitance changes during stretching, often requiring re-tuning mechanisms [23], [24].

Modular coil systems with rigid housings have been introduced by MR equipment vendors [25], described in patents [26] and research publications [27]. More recently, in work on flexible coil design the idea of modularity was picked up: A patent describes modular local coil arrangement [28] and Özen *et al*. elaborate the importance of modular coil arrays and illustrate concepts but only present single-channel data [29]. For 7 T MRI, a modular array with 3.5 cm diameter loop elements for ex-vivo imaging was proposed by Urayama *et al.* [30]. Our group examined the feasibility of modular 3 T coil array design in conference abstracts, showing phantom and preliminary bench data [16], [17]. Proof-of-concept in vivo MRI with 7–8 coil elements individually attached to a face-mask or a t-shirt at the location of the shoulder in a modular fashion have been presented in recent work [31].

To the authors’ knowledge, to date, no complete hardware implementation for a 3 T coil system which exploits the advantages of both concepts, modularity and flexibility, has been benchmarked against the commercially available coils.

On this basis, the goal of our work was to provide a versatile solution for a customizable assembly of surface coil arrays allowing for a FOV coverage tailored to the application. This leads to a single coil array system suitable for a multitude of applications, thereby eliminating the need for multiple expensive coils for each anatomical region. In addition, we aimed at enhancing image quality with close-fitting design and at enabling parallel imaging with acceleration rates higher than or at least equal to those achievable with rigid commercial coils. Light-weight and flexibility are factors which ideally improve patient comfort and coil handling as compared to bulky rigid coils.

In this study, we propose a modular system of flexible lightweight 4-channel coaxial RF coil arrays for 3 T MRI (“ModFlex”). We present the hardware implementation, extensive bench tests, phantom and first in vivo MR measurements with a system of up to 16 receive-channels for different use cases. We compare its SNR and parallel imaging performance to dedicated commercial reference coils in neck, ankle, spine and hip imaging at 3 T.

## 2 Materials and methods

### 2.1 Coil features

**Figure 1:**
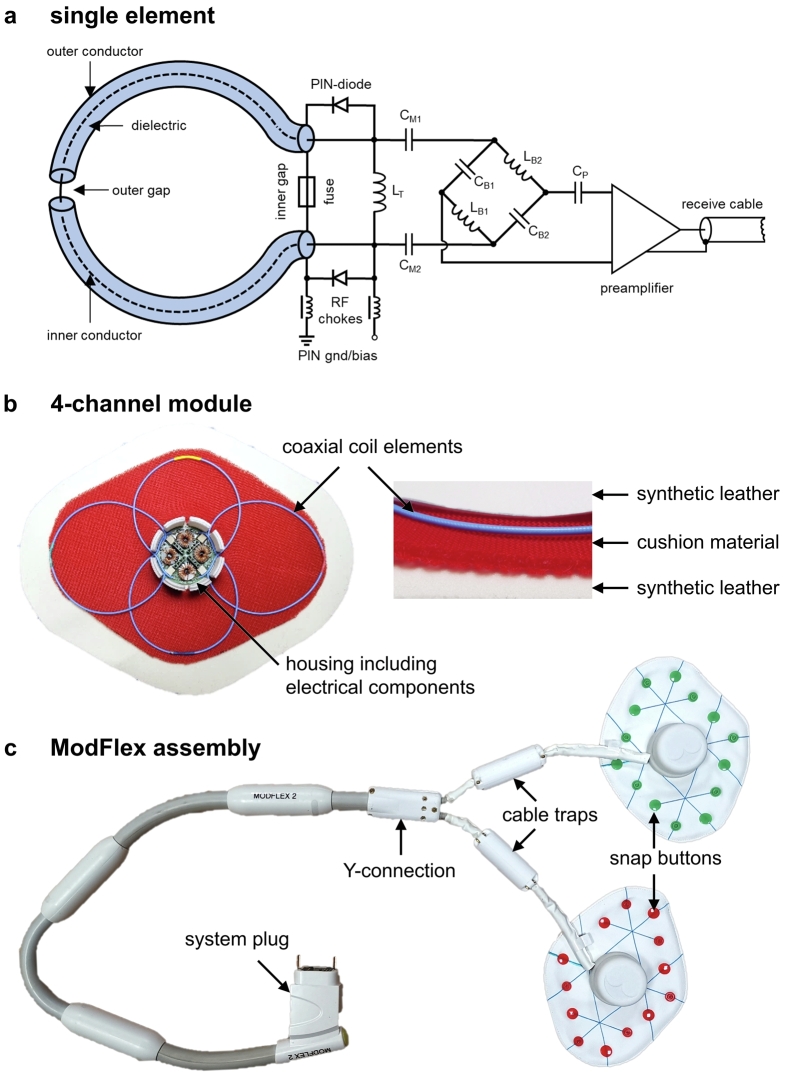
**Coil design.** a) Electrical circuit diagram for a single coaxial coil element, b) details on the components of a 4-channel module, c) photograph of an 8-channel ModFlex coil array connected to the MR system plug via a Y-connection of two 4-channel modules with a custom 3D-printed housing.

Fig. 1 shows an 8-channel ModFlex consisting of two identical flexible coil modules [32]. In each module, four single-gap coaxial coils [8], [14] with 8 cm loop diameter and ≈1.4 mm cable thickness [16] (Molex 047SC-2901, Lisle, Illinois, USA) are embedded in three textile layers. The coaxial coils are operated close to their self-resonance, leading to a homogeneous current density distribution at their outer conductor [8].

A compact biocompatible 3D-printed housing (laser-sintered polyamide PA2200) holds two stacked printed circuit boards with all electrical components for fine-tuning to the Larmor frequency of 123.2 MHz (L_T_), matching (C_M1_, C_M2_) and active detuning with a pair of PIN (positive intrinsic negative) diodes (MACOM, Lowell, MA, USA) and RF chokes (Coilcraft, Cumbernauld, UK). The coils were fine-tuned and matched on a volunteer subject. The broadband active detuning circuitry in each receive-only coil element has the function to block induced currents on the ModFlex coil during RF transmission with the body coil. As an additional safety measure, a fuse with 315 mA current rating is located in the outer conductor at the coil port in case the coil’s active detuning switching network should fail. If the fuse interrupts the connection of the outer conductor, i.e. creates an open circuit, the resonant transmission line behavior will be eliminated, preventing the formation of high currents on any coil conductor. To avoid induced currents on the cable shields, a floating shield current suppression trap [33] (>25 dB attenuation at 123.2 MHz) is placed on each module’s cable strand.

Neighboring coils are geometrically decoupled by overlap which was found to be optimal at 6 cm (same as for conventional coils). Non-overlapping coil elements are only decoupled by the preamplifier decoupling circuit. “Reverse” preamplifier decoupling [14] is implemented with a balun (C_B1_, L_B1_, C_B2_, L_B2_) and phase shift (C_p_) network and on-coil low-noise preamplifiers (28 dB gain, 0.55 dB maximum noise figure, MwT, Fremont, CA, USA).

Two textile layers – cushion and synthetic leather material (TG1019250 and KL1100001, StoffPalette, Donaueschingen, Germany) – at the patient side of the coil create enough insulation between coil conductors and the patient, determined by the dielectric strength of the materials from their product data sheet and creepage distances according to IEC 60601-1. Each module is routed to a Y-connection, linking two modules to a single MR system cable (TIM 3G, Siemens Healthineers, Erlangen, Germany), connected to the coil socket on the patient table via adapters (TIM Coil Interface 3T #10500088, Siemens Healthineers, Erlangen, Germany). The “on-patient” weight, i.e., the coil weight without system cables, which are usually placed on the patient bed, of <0.5 kg per 8-channel coil array ensures patient comfort and convenient coil handling. Straps with snap buttons on both ends can be connected to the modules for coil fixation if necessary.

**Figure 2:**
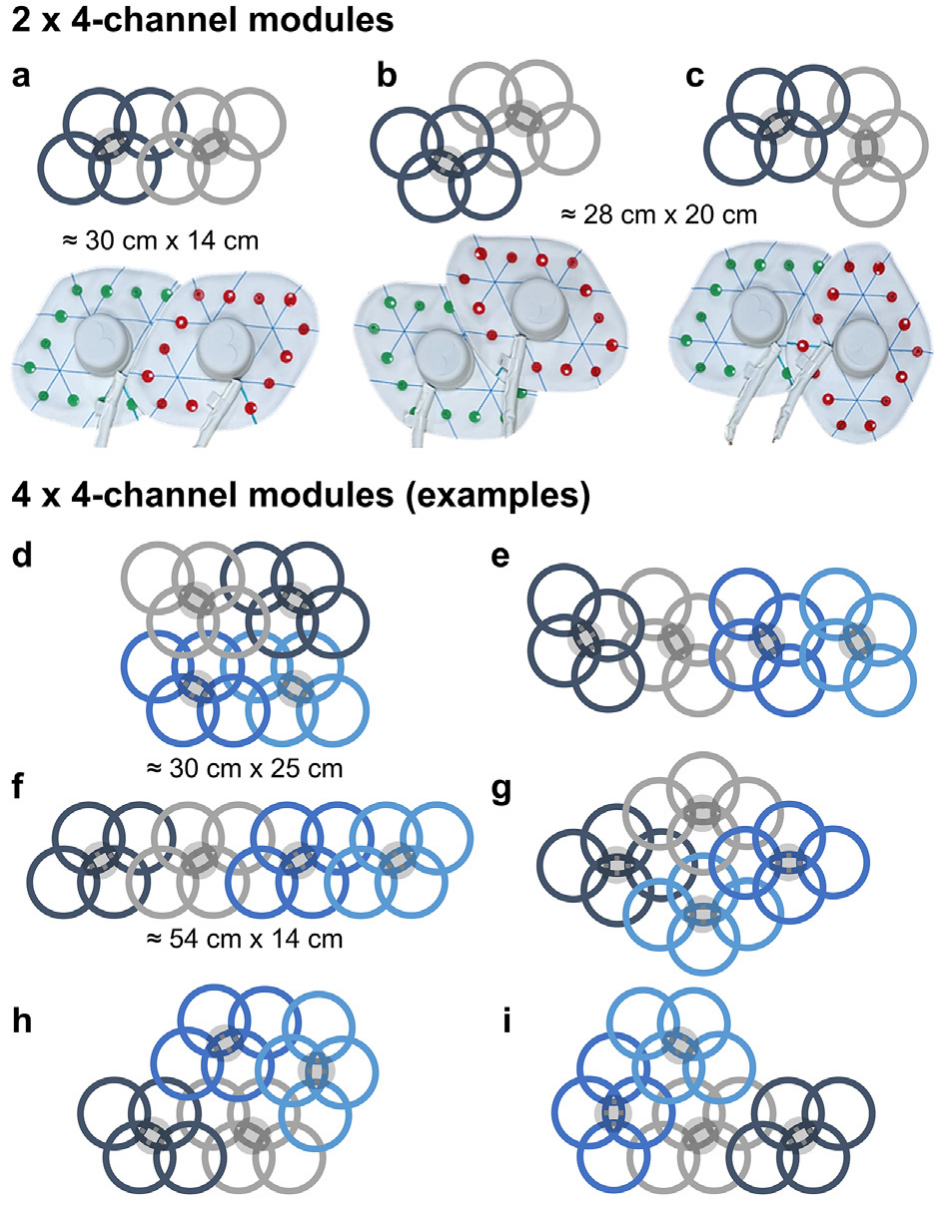
**Module configuration possibilities.** The schematics show different possible module configurations for 8-channel and 16-channel arrays with either 2 (a-c) or 4 modules (d-i).

**Figure 3:**
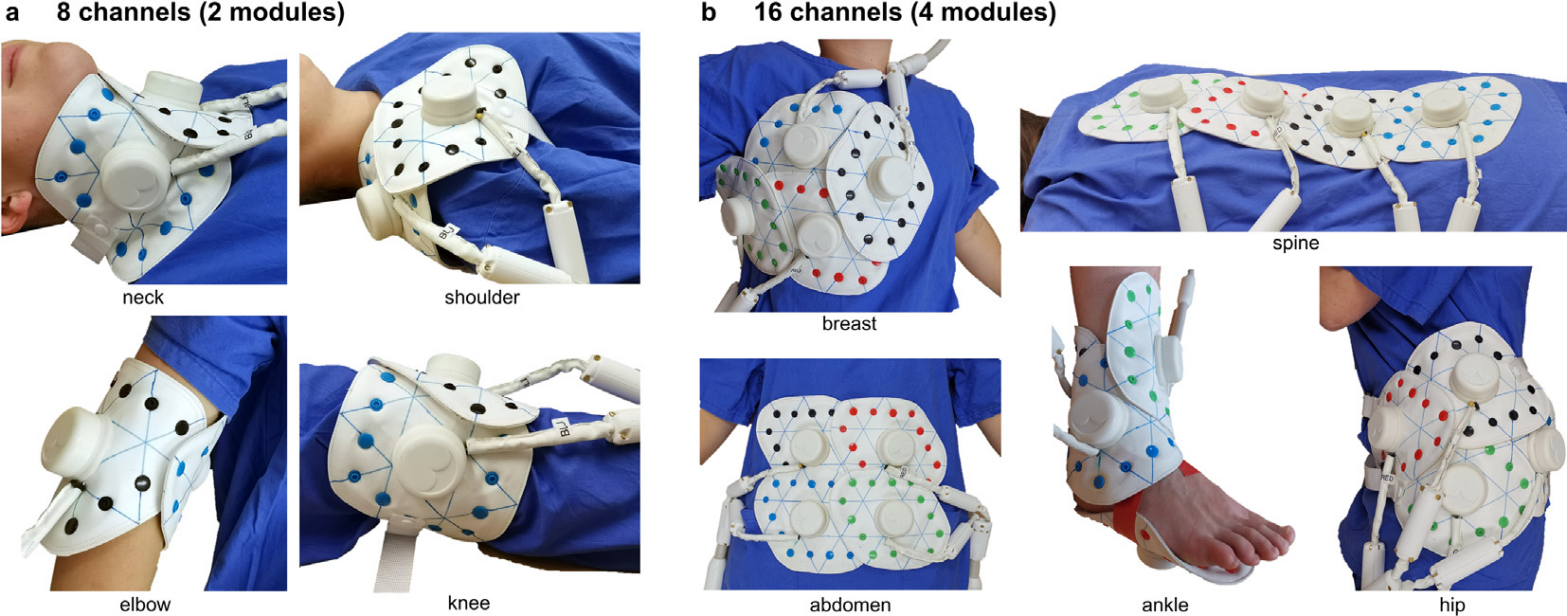
**Overview of ModFlex application examples.** 8-channel examples are assembled according to Fig. 2a, 16-channel examples are assembled as in Fig. 2h (breast), 2f (spine), 2d (abdomen), 2g (hip), 2a+c (ankle). Straps with snap buttons on both ends (not shown here) can be connected to the modules for coil fixation if necessary. Continuous lines on the upper synthetic leather layer of the coil indicate correct module assembly, i.e., ensuring geometric inter-element decoupling and mechanical stability.

**Figure 4:**
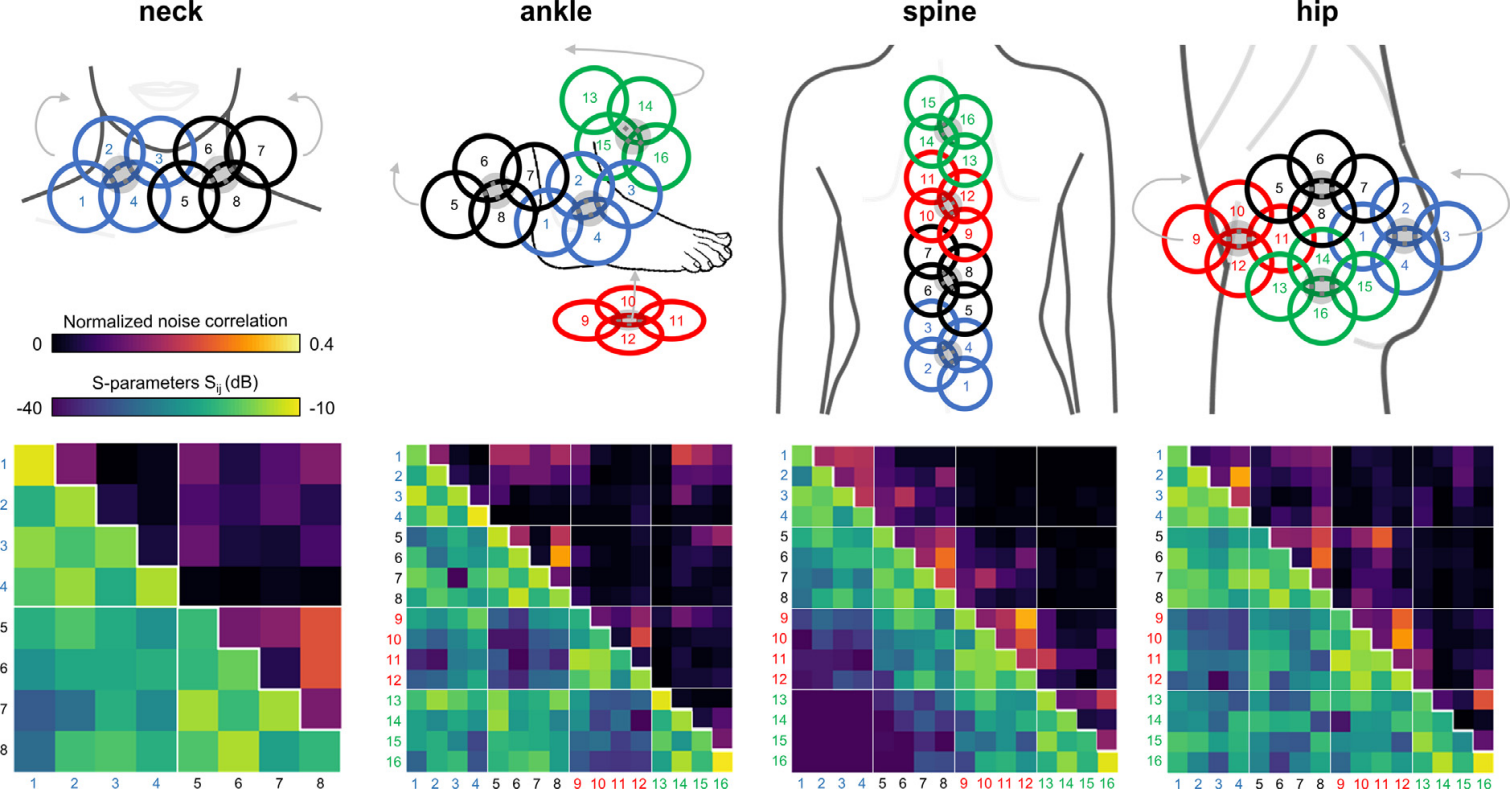
**Normalized noise correlation and S-parameter matrix measured in vivo (neck, ankle, hip and spine) with 8-channel and 16-channel ModFlex coils.** Module configurations and coil positioning are shown in the first row. The bench measurement setup corresponds to the coil positioning used during MRI. Numbers next to the S-parameter and noise correlation matrix indicate the Rx channel number; the colors identify the different 4-channel modules. Thin white lines in the matrix mark the transition between individual 4-channel modules. The diagonal matrix elements of the normalized noise correlation are 1 and are therefore omitted.

To sufficiently cover a desired FOV depending on the imaging application, all coil modules can be attached together via plastic snap buttons in different configurations. Some example configurations for various use cases are shown in Fig. 2. Button colors enable the identification of modules at the MR console and (de-)activation of those 4-channel modules needed to image the target FOV. In this study, the coil configurations used during phantom tests were *a* and *d* (on the abdominal region of a torso phantom). For in vivo experiments, the 8-channel ModFlex in configuration *a* was used for neck imaging and different 16-channel configurations were used for spine, hip and ankle imaging (*f*, *g*, *a*+*c* and separate module, see also Figs. 3 and 4).

Fig. 3 illustrates how the coil positioning can be realized on a volunteer. Other possible application examples with one or two ModFlex coils (8 or 16 coil channels) include e.g., the breast, shoulder, knee, elbow or abdomen.

During preliminary studies [34], [35] the proposed coaxial coil unit was compared to a custom-built 4-channel surface loop coil array of the exact same dimensions fabricated out of stranded copper wire. The comparison on the bench revealed similar overlap and preamplifier decoupling performance [34]. From phantom MR measurements [35] it could be concluded that the SNR in a cylindrical ROI underneath a 4-channel coaxial and stranded copper wire array is comparable. Additionally, we demonstrated the achievable SNR gain on phantom [17] when using an 8-channel ModFlex coil (≈30 cm × 14 cm) compared to a commercial 4-channel coil array of similar dimensions (36.6 cm × 17.4 cm, “Flex 4 small”, Siemens Healthineers, Erlangen, Germany). The above-mentioned experiments on phantom allowed for a first validation of the coil technology during the development phase. In this work, experiments were focused on the comparison of application-ready 8-channel or 16-channel ModFlex coil arrays to commercial product coils for various anatomical targets in vivo.

### 2.2 Bench tests

Bench tests were performed with a vector network analyzer (E5071C, Keysight Technologies, Santa Rosa, USA). S-parameters were measured in different 8- and 16-channel configurations in vivo with ModFlex placed on the neck, ankle, spine or hip to determine matching levels (S_ii_) and overlap decoupling efficiency (S_ij_). A dual-loop probe [36] was used to characterize the active detuning and preamplifier decoupling performance via S_21_ difference (ΔS_21_) measurements at the Larmor frequency. For the active detuning efficiency, ΔS_21_ between the tuned and detuned state of the coil was measured. ΔS_21_ between a coil with preamplifiers plugged (and powered at 10 V, 25 mA) and unplugged (and the coil 50 Ω-terminated instead) was measured to evaluate preamplifier decoupling. Further, *Q*-factors were measured for an unloaded and loaded coaxial coil element of the proposed unit, again using the double-loop probe (S_21_ measurement).

### 2.3 Phantom MR experiments

All MR experiments were carried out on a 3 T MR scanner (Prisma Fit, Siemens Healthineers, Erlangen, Germany). Preliminary MR tests concerning patient safety, the receive performance and the interaction between transmit and receive coils were carried out on phantoms prior to using the coil in vivo.

A torso phantom filled with gel mimicking tissue electrical properties (*σ* = 0.60 S/m, *ε*_r_ = 62) was used.

To characterize the efficiency of both, the active detuning circuitry and cable traps, flip angle (FA) maps were acquired with the body coil using a saturated turbo fast low-angle-shot (TurboFLASH) sequence [37] (2.5 × 2.5 mm^2^ in plane resolution, 5 mm slice thickness, repetition/echo time (TR/TE) = 6460/1.97 ms, FOV = 240 × 320 mm^2^, 490 Hz/px). For the purpose of quantifying B_1_ distortions due to the presence of the receive coil, FA maps were measured for three different setups: 1) with the torso phantom alone as a reference, 2) with a detuned 8-channel ModFlex coil (configuration *a*), and 3) with a detuned 16-channel ModFlex coil (configuration *d*) positioned on the torso phantom.

Furthermore, the maximum surface temperature was monitored at different locations on and below the coil modules using thermo-optic probes (OmniFlex System, Neoptix, Canada) during 1) a GRE pulse sequence with high specific absorption rate (SAR) and fast switching of the PIN diodes (≈17 minutes acquisition time) and 2) an echo-planar imaging (EPI) sequence with high gradient eddy currents (≈7 minutes acquisition time). The “high SAR”, i.e. high RF power output, GRE sequence operated at the shortest possible TR of 7 ms with 100 % allowed SAR for the set dummy patient weight and height as indicated by the scanner calculations and as recommended by the IEC guideline 60601-2-33. The EPI sequence was acquired with 0.65 ms echo spacing. Temperature measurements were consecutively performed with the 8-channel ModFlex coil configuration *a* and 16-channel configuration *d*.

To assess potential RF interferences, a noise spectrum was acquired using the body coil with a bandwidth of ±500 kHz around the Larmor frequency with and without the ModFlex coil present.

### 2.4 In vivo MR experiments

The in vivo study was authorized by the local Ethical Committee of the Medical University of Vienna (nr. 2137/2021) according to the Declaration of Helsinki and was approved by the Austrian authorities in compliance with the Medical Device Regulation 2017/745 Article 62. Informed consent was obtained from all volunteers.

Four different body parts in healthy subjects (all male, age 25-34 years, body mass index 20-26 kg/m^2^) were chosen to evaluate the ModFlex coil performance during MRI experiments: the neck (8-channel ModFlex), the right ankle, the spine and the left hip joint (16-channel ModFlex).

The following commercially available coils were taken as a reference for each respective anatomy: a rigid head/neck coil with 38 out of 64 coil channels activated, a rigid 16-channel foot/ankle coil, a rigid 32-channel spine coil, and a semi-flexible multi-purpose 18-channel coil placed around the hip (all coils from Siemens Healthineers, Erlangen, Germany; product names “Head/Neck 64”, “Foot/Ankle 16”, “Spine 32” and “Body 18”).

Fig. 3 demonstrates the ModFlex coil positioning on a volunteer, with the coil directly placed on or wrapped around the target anatomy. In ankle MRI, ModFlex allows for a more relaxed ankle position which is not fixed to a 90° angle as it is the case with the commercial coil. For spine MRI, the ModFlex coil was placed inside a styrofoam plate integrated in the patient table and imaging was performed in supine position. In general, also prone positioning is possible, e.g. for patients incapable of lying in supine, with the drawback of potentially introducing motion artifacts (breathing, involuntary body motion). Neck and hip imaging were performed in supine position with fixation straps form-fitting the coil to the volunteer. With product coils, positioning was done as intended by the manufacturer and the semi-flexible “Body 18” coil was wrapped as closely as possible around the volunteer’s hip in supine position, comparable to the ModFlex coil setup with a part of the coil placed under the hip, i.e., between the patient table and the volunteer.

The MRI protocol consisted of sequences targeting the technical coil performance evaluation which were measured per volunteer and per coil (ModFlex and corresponding reference coil): after a localizer scan, a 3D T_1_-weighted gradient echo (GRE) sequence with 1.6 × 1.6 mm^2^ (ankle and spine) or 2.1 × 2.1 mm^2^ (neck and hip) in plane resolution (TR/TE = 3.7/2.5 ms, 3° flip angle, 2.0-2.2 mm slice thickness, 1490-1570 Hz/px), and a noise-only scan (without any transmit RF pulse or imaging gradients) were acquired. These data were used for SNR evaluation in a volume of interest and to determine the noise correlation, i.e., coupling between individual coil channels. In parallel imaging, the so-called geometry-factor (*g*-factor) which depends on the signal correlation between individual coil channels and is spatially variable, decreases the SNR in addition to the square root of the acceleration factor *R*. Thus, to assess the coils’ parallel imaging capabilities, 2D T_1_-weighted GRE sequences for g-factor calculation with 1.4 × 1.4 mm^2^ (neck) or 1.6 × 1.6 mm^2^ (ankle, spine, hip) in plane resolution (TR/TE = 50-805/2.9-4.9 ms, 30° flip angle, 2.5-3 mm slice thickness, 1420-1530 Hz/px) were measured. Slice acquisition planes were chosen to simulate acceleration in different phase encoding directions, i.e., anterior-posterior (AP), head-foot (HF) or left-right (LR), as implemented in clinical imaging protocols. We acquired sagittal slices of the neck (HF acceleration), the ankle (AP acceleration), and the spine (HF acceleration), and coronal slices of the hip (LR acceleration).

In addition, standard imaging sequences were acquired with diagnostically relevant contrasts, and the highest resolution possible within clinically reasonable acquisition time (1:15-5:32 min). The “prescan normalize” option was applied for signal intensity gradient correction, which especially facilitates image windowing when using small surface coil elements because of the high SNR in superficial areas. Sequence details and imaging parameters for each body part are given in Table 1.

**Table 1:** 2D MR sequence parameters used during in vivo measurements with an 8- or 16-channel ModFlex coil and reference coils for different anatomical target regions.

**target area**	**neck**	**ankle**		**spine**		**hip**	
**sequence type**	T1-w. TSE	T1-w. TSE	PD-w. fat sat.	T1-w. TSE	T2-w. TSE	T1-w. TSE	PD-w. SPAIR
**imaging plane**	sagittal					coronal	
**in-plane resolution (mm^2^)**	0.57 × 0.57	0.25 × 0.25	0.39 × 0.33	1.11 × 0.83	0.90 × 0.68	0.56 × 0.56	0.63 × 0.56
**matri**x **size**	384 × 384	640 × 640	381 × 448	450 × 384	546 × 448	320 × 320	288 × 320
**slice thickness (mm)**	3						
**slices**	20	22	20	15		23	20
**averages**	1			2	1	2	
**GRAPPA acceleration factor (phase encoding direction)**	2 (HF)	3 (AP)	-	2 (HF)	-	2 (LR)	2 (HF)
**TR/TE (ms)**	729/9.5	1020/17	3000/36	650/9.5	3500/103	710/12	3930/39
**BW/px. (Hz)**	255	260	248	250	248	252	150
**flip angle (°)**	150	140	150	150	160	120	180
**acquisition time (mm:ss)**	01:15	03:05	04:05	03:07	04:14	03:50	05:32

T1/T2-w – T1/T2-weighted, PD – proton density, TSE – turbo spin echo, fat sat – (standard) fat saturation, SPAIR – spectrally attenuated inversion recovery fat saturation, GRAPPA – GeneRalized Autocalibrating Partial Parallel Acquisition, HF – head-foot, AP – anterior-posterior, LR – left-right, TR/TE – repetition/echo time, BW – bandwidth, px – pixel.

### 2.5 Data post-processing

In MATLAB 2021b (The Mathworks, Inc., Natick, MA, USA) we calculated SNR maps based on the pseudo multiple replica method from 3D in vivo GRE and noise only data, and extracted the normalized noise correlation matrix [38]. A GRAPPA reconstruction [39] framework and 2D GRE together with noise-only data was used for the calculation of SNR maps with a fully sampled or undersampled *k*-space, and thereof, *g*-factor maps which were smoothed with a 3 × 3 mean filter. Acceleration factors *R* ranging from 2 to 6 and different slice orientations for each anatomical target region were simulated and maximum and mean *g*-factors extracted for the slice covering the regions of interest (ROIs). To visualize deviations between FA maps acquired with the body coil or a detuned ModFlex coil placed on the phantom, relative FA difference maps were calculated.

To determine mean SNR differences between ModFlex and the reference coils, 6 anatomical targets, i.e. 3D ROIs, were defined and manually segmented using 3D Slicer v4.11. [40], [41]:

• ROI 1 covers the soft neck tissue region anterior to the cervical spine ranging from the tongue to the collarbone,
• ROI 2 covers the cervical vertebrae (C1-7) and one thoracic vertebra (Th1) including intervertebral discs,
• ROI 3 covers the right ankle ranging from the lower tibia/fibula (≈2 cm) to the Lisfranc joints (cut off at metatarsal bones),
• ROI 4 covers 16 thoracic, lumbar and sacral vertebrae (Th3-S1) including intervertebral discs,
• ROI 5 covers the spinal cord along these vertebrae, and,
• ROI 6 covers the femoral head of the left hip.

## 3 Results

### 3.1 Bench tests

In Fig. 4, S-parameter matrices are graphically displayed with the corresponding module configuration and channel numbering. The worst case matching level, i.e. maximum S_ii_ (depending on the application) was -11.5 dB (neck), -10.2 dB (ankle), -11.3 dB (spine), -10.7 dB (hip). Mean matching values were -17.4 dB (neck), -15.6 dB (ankle), -16.6 dB (spine), -18.8 dB (hip). Maximum S_ij_, i.e. worst case decoupling was -15.0 dB (neck), -13.4 dB (ankle), -16.7 dB (spine), -13.2 dB (hip). Stronger coupling, i.e. higher S_ij_ (and also noise correlation), can generally be observed for next-nearest neighboring elements without optimal overlap conditions, e.g. coil element 1 and 3 in configuration *a* in neck imaging. All S-parameters (and noise correlation values, detailed in the Results Section 3.3) are within a range ensuring sufficient element matching and inter-element decoupling. As expected, it can be observed that the general level of coupling between elements depends on the overall arrangement of the coils, ranging from higher coupling for the more compact layouts (ankle, hip) to lower coupling in the most elongated layout (spine).

The measured S_21_-difference between tuned and detuned state of all coils at Larmor frequency was >25.0 dB and the preamplifier decoupling efficiency was >11.4 dB. The coaxial coil elements used in each 4-channel module have a *Q*_unloaded_ of ≈95 and a *Q*_loaded_ of ≈35, resulting in a *Q*-ratio of ≈3, proving a sample-noise dominated measurement condition.

### 3.2 MR phantom experiments

**Figure 5:**
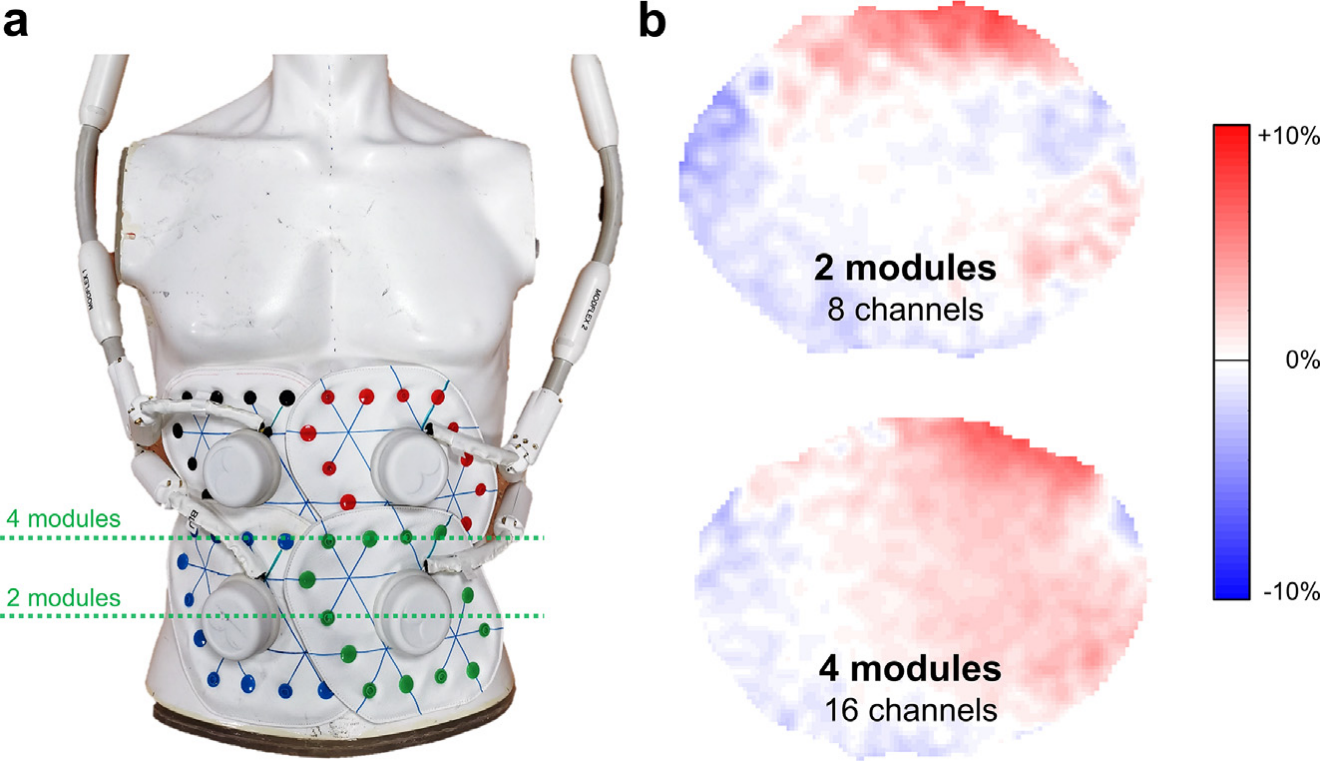
**Decoupling efficiency between body (transmit) and ModFlex (receive) coil**. a) A gel-filled torso phantom was used to measure flip angle maps and calculate b) relative flip angle difference maps for the 8-channel and 16-channel ModFlex coil with either 2 or 4 modules present compared to the body coil alone. The positions of the axial slices are indicated by green dashed lines.

FA difference maps calculated from acquisitions with and without the ModFlex coil present are shown in Fig. 5. The maximum of 7% flip angle change over the whole phantom with the ModFlex coil present as compared to the body coil alone is regarded as sufficient in terms of decoupling of the ModFlex from the body coil.

Heating tests revealed a maximum temperature increase of 2-3° C for all probe positions, i.e., always below the IEC 60601-2-33 and 60601-1 limits.

The body coil’s noise spectra were not affected by the presence of the ModFlex coil.

### 3.3 In vivo imaging

Normalized noise correlation matrices are shown together with S-parameter matrices in Fig. 4: maximum values of 0.24 (neck), 0.32 (ankle), 0.33 (spine), 0.32 (hip) indicate sufficient inter-element decoupling.

**Figure 6:**
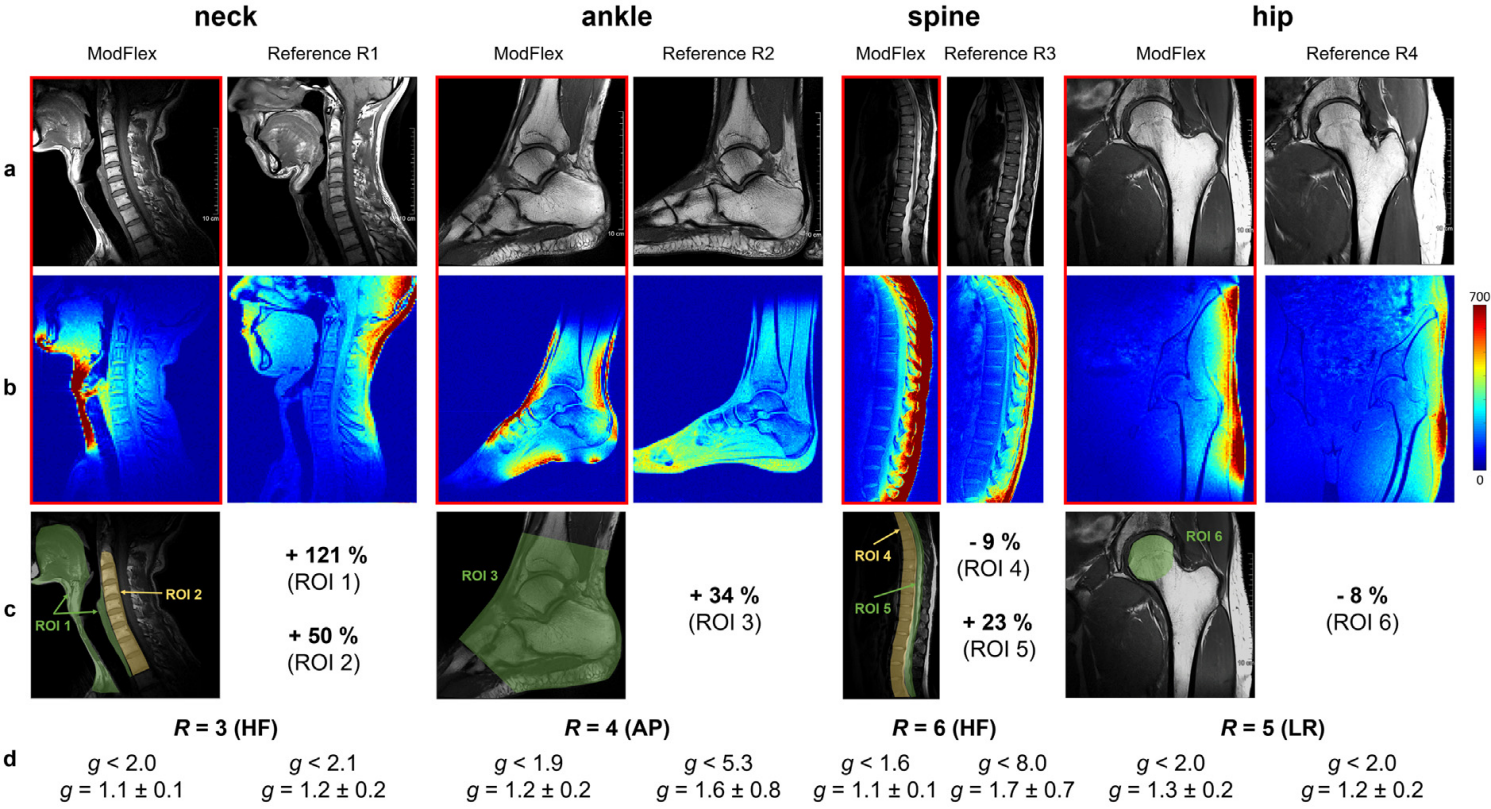
**In vivo SNR and parallel imaging performance evaluation**. a) T1-weighted MR images of the neck, right ankle, spine and left hip acquired with a flexible 8- or 16-channel ModFlex coil compared to a commercial reference coil, b) SNR maps calculated from low resolution GRE and noise-only MR scans, c) Illustration of segmented 3D ROIs defined for SNR evaluation per body part and relative SNR difference (in %) between the reference and ModFlex coil in the respective ROIs. d) *g*-factor evaluation at the cut-off acceleration factor for ModFlex (*g* < 2) and the mean and standard deviation of the *g* value in the respective slice shown in a)-c) covering the ROI(s).

The T1-weighted MR images in Fig. 6a, SNR maps in Fig. 6b and the calculated relative SNR difference between reference coils and ModFlex are given in Fig. 6c. It is demonstrated that the mean SNR in the target 3D ROIs with ModFlex is comparable to or higher than with the respective reference coils: In neck MRI, a 120 % SNR gain in soft tissue from tongue to collarbone and 50 % SNR gain in the cervical spine was achieved. 34 % SNR gain was found in ankle MRI between lower tibia and Lisfranc joints. A slight SNR loss of 9 % SNR in the spine (Th3-S1), a 23 % SNR gain in the spinal cord, and 8 % SNR loss in the femoral head were measured.

Fig. 6d summarizes the parallel imaging performance of ModFlex compared to reference coils with different GRAPPA acceleration factors and phase encoding directions for all body parts investigated in this study. Assuming *g* < 2 as a threshold to limit *g*-factor artifacts in the region of interest, we found high cut-off acceleration factors that can be used with the ModFlex coil:

*R* ≤ 3 in HF direction for the neck, *R* ≤ 4 in AP direction for the ankle, *R* ≤ 6 in HF direction for the spine, and *R* ≤ 5 in LR direction for the hip. Overall, in neck and hip imaging, *g*-factor artifacts are comparable between ModFlex and reference coil at the cut-off acceleration factor. For spine and ankle imaging, we show that acceleration possibilities with the ModFlex coil are higher (i.e., *g*-factors are lower) than with the reference coil. Therefore, ModFlex enables faster imaging in cases where the SNR is not already penalized too much by the factor of $\sqrt{R}$ reduction in parallel imaging. SNR maps reconstructed based on undersampled k-space data revealed that even with a reduction by *g*$\sqrt{R}$, the SNR in the target ROIs achieved with ModFlex remains high compared to reference coils. This statement exempts hip imaging as, here, the SNR in the region around the femoral head and *g*-factors are comparable between coils.

**Figure 7:**
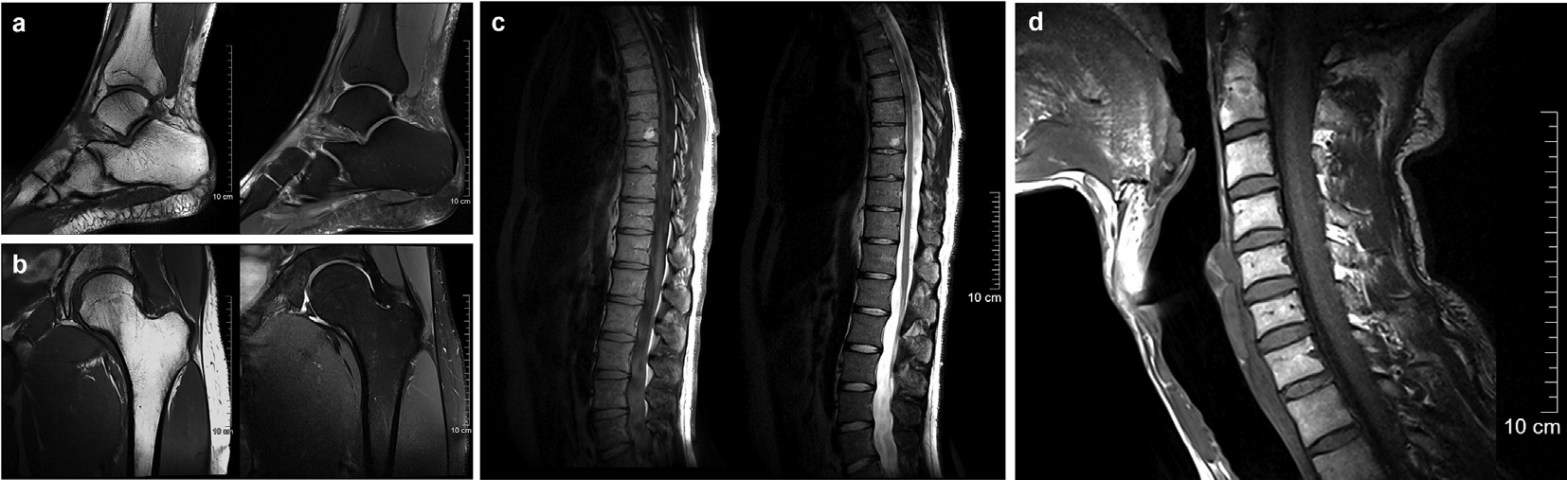
**In vivo MR images acquired with an 8- or 16-channel ModFlex coil** of the a) right ankle: T1-weighted (left) and PD-weighted (right) image, b) left hip: T1-weighted (left) and PD-weighted (right) image, c) spine: T1-weighted (left) and T2-weighted (right) image, d) neck: T1-weighted image. MR sequence details and parameters are given in Table I.

The versatility of ModFlex is further demonstrated by the acquired in vivo images with different clinically relevant pulse sequences in Fig. 7.

## 4 Discussion

In this work, we have developed ModFlex, a lightweight and ultra-flexible modular coil array concept and demonstrated its performance in a broad range of applications.

The coil’s robustness in terms of mechanical stability and electrical performance for different use cases was not compromised by the modularity and flexibility of the coil design approach. The coil can be considered as lightweight because of both, the conscious choice of very small components (e.g., PIN diodes, preamplifiers, connectors, small PCBs), and the optimization of housing material (e.g., design of thin but still robust walls) and textile layers. The exact weight of the 16-channel ModFlex coil is 0.6 kg without cabling or 0.8 kg including cabling until the Y-connection, cable traps and the Y-connection itself, which are sometimes also placed on the patient. Both values are well below the weight of the “Body 18” coil (Siemens Healthineers, Erlangen, Germany) of 1.1 kg [42] without any cabling.

The high achievable local SNR was shown to be particularly beneficial for ankle, spinal cord and soft neck tissue imaging, while for hip and thoracic vertebrae imaging larger coil elements would be slightly better suited. This could possibly be compensated by combining more than one 16-channel ModFlex wrapped around the area of interest or by fabricating a similar flexible coil with larger individual loop elements. Despite the slight SNR loss at larger depth in spine and hip images, the ModFlex coil’s SNR in the ROI is sufficient for a large range of subjects.

As with all small surface coils, there is a rather steep image intensity gradient from the location of the coil elements towards the inside of the subject. This aspect could potentially confuse readers at a first look and interfere with diagnosis, or lead to the assumption of lower SNR in the ROI due to inappropriate image windowing. This issue can be alleviated by using the scanner-integrated intensity correction and could be further optimized in post-processing [43].

ModFlex coils could in principle be combined with existing commercial coils, e.g. the spine coil inside the patient bed together with a 16-channel ModFlex coil placed on the abdomen, but such combinations are currently limited by vendor constraints.

The proposed 16-channel ModFlex coil could encounter limits if larger areas of interest or larger patients need to be imaged. This can be overcome by the modularity of the system which is easily scalable up to the Rx channel count enabled by the scanner system. In related work [44], the flexible coil module concept was exploited for the design of a 28-channel wearable MR coil vest for supine breast MRI.

Furthermore, the usability of the coil design is currently being explored for the fabrication of an RF coil head cap [45]. In addition to a larger FOV coverage, a higher individual coil channel count can also lead to higher acceleration possibilities, e.g. if coils are placed around an area of interest. In the clinical images shown in this article, we closely followed the pulse sequence parameters set in standard product sequences. We did not attain the acceleration limits with a ModFlex coil and always stayed with acceleration factors where simulations showed *g*-factors well below 2. Consequently, MR sequences could further be optimized for ModFlex coils, taking advantage of the very high SNR, e.g. in neck or ankle imaging, to significantly reduce measurement time while achieving the same SNR as commercial coils.

In the present study, we tested the ModFlex coil performance only in a small study population as the focus lies on the technical evaluation in a few in vivo measurements in the frame of a pilot study. To investigate whether the findings are representative for a larger population, a clinical study with 40 subjects is planned. The aim of this future study will be to highlight even more the usability of this coil for different body sizes, shapes and parts by choosing a more diverse subject cohort and also evaluate the effect on patient comfort.

In conclusion, the presented flexible modular coil array system improved SNR for 4 out of 6 and showed similar SNR in 2 out of 6 investigated anatomical target regions in neck, ankle, spine and hip imaging as compared to commercial coils. We demonstrate the ModFlex coil system’s high parallel imaging performance, partly outperforming standard coils in different 3 T MRI use cases. In clinical practice, the multi-purpose design principle could benefit sites with limited coil equipment, make the coil setup more convenient and improve patient comfort. The adaptability of the coil is especially useful in applications where patient size and shape show strong variations and image quality would be impaired when using standard rigid instead of form-fitting RF coils. Furthermore, ModFlex is a versatile tool for niche applications where no dedicated coils are available or standard coils limit the imaging possibilities.

## Funding

This research is supported by the Austrian Science Fund (FWF project I-3618 “BraCoil” and project P-35305 “AquaLactEMISM”), the Agence Nationale de Recherche (ANR): ANR-17-CE19-0022, and the CITRUS project that has been funded by the European Union’s Horizon Europe research and innovation programme under Grant Agreement no 101071008.

## Data Availability Statement

The Data Clearing Committee of the Medical University of Vienna has restricted data sharing because data cannot be completely de-identified. Data can be made available to all interested researchers upon request to the data clearing committee of the Medical University of Vienna (datenclearing@meduniwien.ac.at) and the corresponding author (elmar.laistler@meduniwien.ac.at).

## Declaration of Competing Interest

The authors declare that they have no known competing financial interests or personal relationships that could have appeared to influence the work reported in this paper.
